# The Left–Right Polarity Puzzle: Determining Embryonic Handedness

**DOI:** 10.1371/journal.pbio.0030292

**Published:** 2005-08-16

**Authors:** William B Wood

## Abstract

Whenever symmetry is broken in nature to yield only one of two equally probable outcomes, there is an intriguing problem to be solved.

Whenever symmetry is broken in nature to yield only one of two equally probable outcomes, whether in physics, chemistry, or biology, there is an intriguing problem to be solved. Physicists from M. and P. Curie to T.-S. Lee and C.-D. Yang puzzled over such phenomena at the atomic level. Organic chemists puzzled over the handedness of molecules for many years after Pasteur showed that grape juice contained only one of the possible right- and left-handed mirror-image forms (enantiomers) of tartaric acid (Figure 1; see Glossary [Box 1]). And biologists continue to puzzle over the handedness of organisms.

**Box 1. Glossary**

**Bilaterian:** having bilateral symmetry.

**Chiral:** having chirality.

**Chirality:** the screw sense (handedness) of a helix. Its mirror image will have the opposite chirality.

**Dextral and sinistral:** right- and left-handed, respectively (referring to laterality or chirality). For chiral structures these terms are absolute (a right- or left-handed screw axis); for laterality, the predominant handedness is often called dextral arbitrarily.

**Enantiomeric:** handed.

**Enantiomers:** the two possible mirror image forms of an asymmetric object (usually applied to molecules) with no bilateral symmetry.

**Handed:** having handedness.

**Handedness:** the difference between two objects that are mirror images of each other, such as the right and left hands.

**Laterality:** the handedness of situs, the arrangement of internal organs (viscera) in the body.

**Situs inversus viscerum:** a condition in which the normal laterality is mirror-image reversed, that is, it develops with opposite to normal handedness. Individuals with this condition may be functionally completely normal.

**Figure 1:**
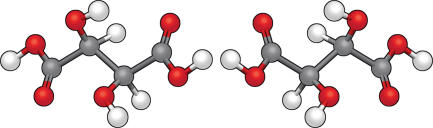
Mirror-Image Symmetry of the Enantiomeric Molecules D- and L-Tartaric Acid Pasteur discovered that a solution of tartaric acid from grape juice (now known to contain only the D form) rotated plane-polarized light, whereas chemically synthesized tartaric acid did not. Pasteur solved this puzzle by showing that the chemically synthesized compound was a mixture of the two forms, which when separated could rotate light in opposite directions.

Why is there a puzzle? The embryos of most, probably all, bilaterians exhibit obvious polarities from head to toe (along the anterior–posterior axis) and back to front (along the dorsal–ventral axis), but they also exhibit less obvious left–right (L-R) differences. That is, although bilaterally symmetrical on the outside, they are L-R asymmetric on the inside. The polarity of the L-R axis determines the laterality of the body plan, for example, whether the human heart will be on the left side or on the right. There are two possible mirror-image forms of the animal body plan, just as there are for tartaric acid, differing only in L-R polarity. Almost without exception, however, the body plans of individuals in any given species develop as only one of the two possible “enantiomers.” This means that at some point during embryonic development, just as in the grape's synthesis of tartaric acid, L-R symmetry must be broken in a unique manner, so that all individuals develop with the same handedness—for example, with the heart on the left. Establishment of asymmetry in embryos is no longer a mystery; we know of several mechanisms by which a cell or a tissue can become asymmetrically polarized. The puzzle here lies in the mechanism of initial choice between two opposite polarities that should be equally probable. We now know that stereospecific synthesis of biomolecules like tartrate comes about because the enzymes that catalyze their synthesis are also stereospecific, handed molecules. As the organic chemist F. R. Japp stated in 1898: “only asymmetry can beget asymmetry” (quoted in [1]). Where does the stereospecific cue in embryonic handedness choice come from?

## L-R Asymmetry Can Be Established Early in Development

Over 100 years ago, H. E. Crampton [2] observed that the handedness, or chirality, of snail shell coiling, dextral (right-handed) for some species and sinistral (left-handed) for others, could be predicted from the handed orientation of the two mitotic spindles prior to second cleavage of the embryo. A. Sturtevant [3], working with a mutation that caused sinistrality in a normally dextral snail species, showed that handedness is controlled by a maternal-effect gene, suggesting that some maternal gene product, incorporated into the oocyte, could influence spindle orientation and the chirality of subsequent shell coiling (although recent work has shown that the early embryonic stages of sinistral mutants are not strict mirror images of the corresponding normal stages [4]). This suggestion was borne out by the finding that sinistral zygotes could be “rescued” to become dextral embryos by injection of cytoplasm from a dextral oocyte [5]; unfortunately, the active substance has not been identified. So snails establish handedness very early; what bilateral symmetry they exhibit must be superimposed later (see Box 2).

**Box 2. Intriguing Questions about L-R Asymmetry and Handedness**

(1) What is the L-R ground state of the embryo? Is it bilateral symmetry, which must be broken to create the L-R asymmetry of the viscera, or is it very early L-R asymmetry, on which later external bilateral symmetry must be imposed?

(2) Why has one handedness been selected by evolution over the other for each species? We know of many examples, from nematodes to humans, showing that when rare mirror-image individuals of the opposite handedness arise, they can be functionally entirely normal [25]. So internal handedness doesn't appear to matter, and yet only one of the two possible body plans is observed in almost all individuals of a species.

(3) Why have animals evolved to be L-R asymmetric internally, rather than bilaterally symmetric as on the outside?

Apparent answers to the first question for at least some animals will come up in the text. About the second and third questions we can still only speculate.

The nematode Caenorhabditis elegans, which exhibits clear laterality of internal organs, has also established handed L-R asymmetry by the six-cell stage [6] and probably earlier (W. B. Wood, unpublished data). The external bilateral symmetry of the animal is imposed during embryonic development by cell signaling [7], which changes the relationship of cell lineage to cell fate on the two sides of the animal to compensate for the physically asymmetric placement of lineally homologous cells in the ectoderm [6].

What about vertebrates? Much has been learned from the study of molecular markers that exhibit L-R asymmetry in their expression (reviewed in [8,9]). In embryos of the frog Xenopus, there is a clear L-R asymmetry of the maternally expressed TGF-β family member Vg1 in vegetal blastomeres as early as the eight-cell stage. Vg1 is seen predominantly on the left side, and injection of Vg1 on the right side leads to random choice of laterality in the resulting embryos (that is, about 50% dextral and 50% sinistral) [10]. More recently, a second, even earlier asymmetry was found at the four-cell stage [11] in localization of the mRNA for a maternally expressed H+/K+-ATPase. Asymmetric localization of this proton pump is important: pharmacological blocking of the ATPase results in randomization of laterality. Recent results with zebrafish [12] and chick [11] embryos have shown ionic potential differences across the midline prior to gastrulation, resulting from asymmetric proton pump activity, and these differences also appear to be required for normal handedness choice. These results suggest that in lower vertebrates, as well as invertebrates, handed L-R asymmetry is established early in embryogenesis, even though morphological L-R asymmetry is not apparent until gastrulation.

Analysis of other signaling molecules in mouse embryos also revealed L-R molecular asymmetries, but not until around the time of early gastrulation, when thousands of cells are present. Subsequent studies showed that these embryos, as well as all the vertebrates mentioned above, have elaborate, presumably homologous asymmetric signaling pathways that function from this point onward to maintain L-R differences on either side of the midline and thereby control laterality of heart looping and asymmetric development of the viscera (reviewed in [9]). (Incidentally, recent work has shown that in vertebrates, too, the symmetry of somite development along the dorsal midline must be superimposed on the underlying pattern of L-R asymmetry by additional signaling [12,13].)

## A Mechanical Polarity Generator

The elucidation of later L-R signaling in mammals does not address the question of when or how L-R asymmetry with the correct handedness is initially established. The first clues to a surprising possible answer to this question came from human, and then from mouse, genetics. Among individuals with Kartagener syndrome, caused by one of several human dynein defects that result in ciliary dysfunction (leading to bronchial problems and male infertility), laterality was found to be randomized; that is, half of these patients exhibited “situs inversus viscerum” (reversed body plan) while the rest had the normal body plan. The *iv* gene in mice, mutation of which also causes randomized laterality, was found to encode a new member of the dynein family, which was named left-right dynein, or Lrd.

The significance of dynein involvement in handedness choice became clear through a remarkable series of discoveries, beginning with the demonstration in mouse embryos that monocilia, present on the node (corresponding to the amphibian Spemann organizer) in early gastrulation and previously thought to be immotile, did in fact beat. Moreover, their beating could move fluorescent beads consistently to the embryo's left, suggesting that they could be providing an asymmetrical cue for handedness determination [14]. Consistent with this view, *iv* mutant mouse nodal cilia appeared to be immotile, and mouse knockout mutations of the *Kif3* kinesin genes, resulting in lack of nodal cilia, also randomized laterality. Artificially created rightward flow resulted in embryos with reversed laterality, and artificial leftward flow with *iv* mutant embryos rescued the mutant defect, strong evidence that the directional flow itself was causative for correct handedness determination [15]. Presumably, the normal direction of the flow was somehow dependant on the intrinsic chirality of the cilia themselves, thus providing a possible physical basis for choice of the correct handedness.

But how the cilia might actually provide such a cue remained an unanswered question until recently. What was being moved? Nonaka et al. [14] originally proposed that the cilia might move an unidentified morphogen, which could trigger asymmetric establishment of the previously defined left and right signaling cascades. Later evidence suggested that the more immotile cilia around the edges of the node could be mechanosensors, containing the polycystic-kidney-disease (PCKD) ion channel protein. It was proposed that these sensory cilia could be activated by fluid flow on only the left side to initiate an observed asymmetric release of Ca^++^, which in turn could activate subsequent asymmetric signaling [16,17].

Another unanswered question was how the nodal cilia could cause leftward flow. Monocilia lack the central-pair microtubules that define the beating direction of “conventional” cilia, and consequently, monocilia move with a uniform rotating motion. Conventional cilia, by contrast, exhibit a back-and-forth beat with defined power and return strokes that can push surrounding fluid in one direction. Rotating cilia should cause local vortices, not a directional flow. Attempts to explain this directionality by the geometry of the nodal depression were unsatisfying [14].

Three recent papers have provided some answers to these questions. What's being moved? Tanaka et al. [18] present evidence that fibroblast growth factor (FGF) in the region of the node stimulates the release of 0.3- to 5-µm vesicles that contain the signaling molecules and possible morphogens Sonic Hedgehog (SHH) and retinoic acid (RA). These “nodal vesicular particles” are swept by nodal cilia to the left edge of the node, where they fragment to release their cargo, which might be the trigger for the previously observed asymmetric rise in local Ca^++^ concentration.

**Figure 2:**
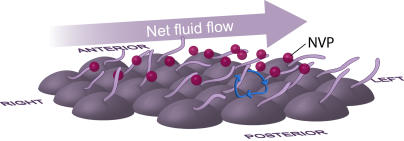
Ventral View of Monocilia on the Mouse Node in Early Gastrulation The diagram shows how clockwise-rotating cilia on the nodal cells can move a fluid suspension of small vesicles containing signaling molecules (nodal vesicular particles [NVPs]; red spheres) toward the left, creating a right-to-left asymmetric gradient across the midline. Key to the cilia's function is the posterior tilt of their rotational axes, as explained in the text. Connection arrows show the trajectory of the tip of one cilium as it rotates.

And leftward flow? A group of fluid dynamicists proposed a simple solution [19]: just tilt the cilia toward the posterior! In this configuration, when the clockwise-rotating cilia stroke to the embryo's right, they will be close to the nodal cell surface, which locally impedes fluid flow, and when they stroke to the left at the top of their arc, they will be away from the surface, where fluid flow is unimpeded. The result will be leftward fluid flow. Reporting in this issue of *PLoS Biology*, Nonaka et al. [20] have used high-speed video microscopy to experimentally validate the predicted posterior tilt. This work accords with a recent independent study [21] and moreover shows that the rightward stroke of each cilium actually brushes along the nodal surface, so that the trajectory of the ciliary tip is a D-shaped rather than a circular arc (Figure 2). As a further test of the fluid dynamic theory, Nonaka et al. [20] built a working model with tilted wire cilia rotating through a viscous medium to approximate the fluid dynamics of the nodal environment. They show that it indeed moved suspended particles in the predicted direction only.

These recent papers provide answers to two major questions about how nodal cilia can cause directional flow and how this, in turn, can initiate L-R asymmetric signaling. While they do not rule out the mechanosensory model, they do show that asymmetric transport of putative morphogens occurs as well. Perhaps both mechanosensors and morphogens are involved in activating subsequent laterality pathways.

## A General Mechanism?

Where does this leave our understanding of handedness choice? There is still a major caveat regarding the mammalian mechanism and its relationship to the presumably homologous mechanisms in other vertebrates. Rotating cilia, transiently present on the node, or equivalent structures in early gastrulation have now been demonstrated or implicated in embryos of mouse, rabbit, chick, zebrafish, medaka fish, and frog [21–23]. In the two mammals and the two fish, this rotation has been shown to move nodal fluid to the left, suggesting that all these embryos, despite very different embryonic and nodal geometries, may use a conserved mechanism for regulating subsequent laterality pathways that is dependent on the inherent chirality of cilia. Still unclear, however, is whether this ciliary rotation is the initial event that breaks L-R symmetry to establish handedness, or whether it serves as an amplifying mechanism for an initial choice that was made earlier in embryogenesis. Most of the vertebrate researchers cited above assume the former possibility, based on experiments showing that directional flow of nodal fluid is both necessary and sufficient for handedness determination. However, if we consider elaboration of L-R asymmetry as a stepwise process or pathway, necessity and sufficiency are to be expected of a downstream component, and they do not preclude the possibility that there are required upstream components as well.

In all but the mammals, L-R asymmetries are known to be present before the node develops. Levin [24] has convincingly reviewed arguments for early laterality cues that could be amplified by the action of nodal cilia. Among these early asymmetries, the potential difference across the midline, in particular, is common to zebrafish, frog, and chick and is necessary for normal development of laterality. There is, to my knowledge, no similar evidence for necessary asymmetries preceding nodal flow in the mouse, but few attempts have been made to find them [24]. Conceivably, the need for earlier cues was lost during the evolution of mammals. But at least the existence of such cues should be rigorously tested in the mouse embryo before assuming they are not present or play no role.

And so, with the possible exception of the mammalian mechanism, the nature of the initial symmetry-breaking cue that dictates correct handedness choice in invertebrates and most vertebrates still eludes us. Parts of the L-R asymmetry picture have become clearer, but there are still several pieces of the puzzle to be put in place.

## Abbreviation

L-R: left–right
